# Exploiting Signal Joint T Cell Receptor Excision Circle to Investigate the Impact of COVID-19 and Autoimmune Diseases on Age Prediction and Immunosenescence

**DOI:** 10.3390/biomedicines10123193

**Published:** 2022-12-09

**Authors:** Amina A. Farag, Taghrid G. Kharboush, Noha H. Ibrahim, Mohamed Darwish, Iman M. Fawzy, Hanaa El-Sayed Bayomy, Dina Saad Abdelmotaleb, Shaza Abdul Basset Abdul Basset, Amal M. Abdel-Kareim, Mohammed Al mohaini, Inas A. Ahmed, Haidy M. Fakher

**Affiliations:** 1 Department of Forensic Medicine & Clinical Toxicology, Faculty of Medicine, Benha University, Benha 13518, Egypt; 2 Department of Medical Microbiology and Immunology, Faculty of Medicine, Benha University, Benha 13518, Egypt; 3 Department of Basic Sciences, College of Sciences and Health Profession, King Saud bin Abdulaziz University for Health Sciences, Alahsa 31982, Saudi Arabia; 4 King Abdullah International Medical Research Center, Alahsa 31982, Saudi Arabia; 5 Department of Rheumatology, Rehabilitation and Physical Medicine Faculty of Medicine, Benha University, Benha 13518, Egypt; 6 Department of Internal Medicine, Faculty of Medicine, Benha University, Benha 13518, Egypt; 7 Mansoura Central Laboratories, Ministry of Health, Mansoura 35511, Egypt; 8 Department of Public Health & Community Medicine, Faculty of Medicine, Benha University, Benha 13518, Egypt; 9 Department of Family & Community Medicine, Faculty of Medicine, Northern Border University, KSA, Arar 73213, Saudi Arabia; 10 Department of Clinical and Chemical Pathology, Faculty of Medicine, Benha University, Benha 13518, Egypt; 11 Department of Zoology, Faculty of Science, Benha University, Benha 13518, Egypt; 12 Department of Basic Sciences, College of Applied Medical Sciences, King Saud bin Abdulaziz University for Health Sciences, Alahsa 31982, Saudi Arabia; 13 Department of Medical Biochemistry and Molecular Biology, Faculty of Medicine, Benha University, Benha 13518, Egypt; 14 Central Laboratory for Research, Faculty of Medicine, Benha University, Benha 13518, Egypt

**Keywords:** age estimation, immunosenescence, autoimmune diseases, COVID-19, *sjTRECs*

## Abstract

Signal joint T cell receptor excision circles (*sjTRECs*) are a promising marker for age estimation and immunosenescence in different ethnic groups. Several limitations are expected to overshadow their use as accurate markers for age prediction. The current study was conducted to determine the influence of immunologic disorders, such as autoimmune diseases and COVID-19, on the accuracy of *sjTRECs* as molecular markers for age estimation and immunosenescence among living Egyptians. Peripheral blood *sjTRECs* level was measured by qPCR in 90 autoimmune patients, 58 COVID-19 patients, and 85 healthy controls. The mean dCt values were significantly (*p* = 0.0002) different between the three groups, with the highest values in healthy subjects, followed by autoimmune and COVID-19 patients. A significant negative correlation was identified between the *sjTRECs* levels and ages in all studied cases. There were significant positive correlations between chronological age and predicted age for healthy individuals, autoimmune, and COVID-19 patients with mean absolute deviations (MAD) of 9.40, 11.04, and 9.71, respectively. The two patients’ groups exhibited early immunosenescence, which was more noticeable among the young adults with COVID-19 and autoimmune patients of age range (18–49 years). Autoimmunity may represent a critical factor impacting the accuracy of *sjTRECs* quantitation for age prediction.

## 1. Introduction

Estimating an individual’s age is a crucial component of forensic science study, which can yield great information pertinent to anthropological, legal, and criminal investigations. Forensic age inference was primarily relied on morphological examination or radiography, and molecular methods were adopted later [1]. Ethnic diversity was found to affect the accuracy of some highly reliable age assessment techniques, causing either over- or under- estimation of dental age [2]. A simple and reliable DNA test based on the quantification of the *sjTRECs* was introduced in 2010 for age estimation from the blood [3]. Additionally, the amount of *sjTRECs* in peripheral blood monocytes (PBMCs) or T cell subsets was used as a particular measure of the quantity of naive T cells and recent thymic emigrant (RTE), which are important markers for evaluating T cell immunity [4].

Immunosenescence is a term used to describe age related involution of the thymus and gradual reduction in T cell number and function. With immunosenescence, people become at higher risk of developing autoimmune diseases, infections, and malignancies [5,6].

Multiple genetic, pathologic, and environmental factors are known to modulate an individual’s immunologic condition, which is closely related to the levels of *sjTRECs.* Hence, various immunologic disorders should be considered during *sjTRECs*’ quantification. The *sjTRECs* concentration in the human body can be affected by the thymic functionality, as well as T cell homeostasis, in the peripheral blood [7,8].

During the search for biomarkers to estimate the chronological age independently from the biological age, the dynamics of the immune system should be considered. It is essential to assess the possible impact of different disease conditions on the process of age estimation in order to expand the approach for clinical applications [3,9]. Additional studies on *sjTRECs* as an age biomarker in healthy, as well as diseased persons of a wide age range, are also required to validate and understand the association between biological and chronological age indicated by this marker [10].

The novel pandemic, COVID-19, has been related to significant health consequences such as those caused by systemic autoimmune diseases. Organ damage in COVID-19 has been shown to be mainly immune-mediated. Moreover, disturbance of self-tolerance to host antigens and development of autoantibodies has been detected in COVID-19 [11].

Accumulating evidence from previous studies suggested that the immunologic disorders associated with acute SARS-CoV-2 infection, and the chronic autoimmune disorders, may impact the pace of ageing in these patients’ groups. Thus, the current study was performed to evaluate utilizing *sjTRECs* gene expression level, as a molecular marker for age estimation and immunosenescence, in the peripheral blood of Egyptians with COVID-19 and different autoimmune disorders versus age/sex-matched healthy controls.

## 2. Materials and Methods

Study Design: this comparative cross-sectional study was conducted between 2020 and 2022 on a convenient sample of 90 patients with autoimmune diseases, 58 confirmed COVID-19 patients, and 85 healthy controls. Pregnant or lactating women, and patients with chronic illnesses other than autoimmune diseases, were excluded from the study. Cases were randomly selected. A single peripheral blood sample was collected from each participant. In COVID-19 disease, an example of acute illness, the sample was collected 24 h after confirmed positive SARS-CoV-2 PCR test. In autoimmune diseases, an example of chronic illness, it was collected from previously diagnosed cases (disease duration ≥ 6 month) during follow up visits, regardless of the stage of the disease activity. The current study was approved by the Benha University Research Ethics Committee, and written informed ethical consents were obtained from the participants or their guardians before enrollment in the study

Study Participants: COVID-19 patients were recruited from the Contagious Disease Control Center (CDCC), Internal Medicine and Pediatric Departments, Benha University Hospital, Egypt. All COVID-19 patients were evaluated using complete medical history taking and full clinical examination. Inclusion criteria: according to the triage protocol of the Ministry of Health and Population, a confirmed COVID-19 case is defined as a patient with COVID-19 infection that has been confirmed by PCR test, regardless of the clinical manifestations. Patients were subdivided into mild (*n* = 35), and severe (*n* = 23). The severity assessment was conducted in all cases according to the Chinese Center of Disease Control (CDC) [12]. These include mild cases of non-pneumonia or mild pneumonia (mild symptoms without dyspnea; respiratory frequency < 30/min; SpO_2_ > 93%; PaO_2_/FiO_2_ ratio ≥ 300 mmHg) and severe cases (dyspnea, respiratory frequency ≥ 30/min, SpO_2_ ≤ 93%, PaO_2_/FiO_2_ ratio < 300 mmHg, and/or lung infiltrates > 50% within 24 to 48 h).

Autoimmune patients were recruited from the inpatients’ ward and the outpatient clinic of the Rheumatology, Rehabilitation, and Physical Medicine Department. All patients were subjected to full medical history taking, as well as general and systemic clinical examination. Patients with different autoimmune disorders include SLE (*n* = 30), RA (*n* = 21), psoriasis (*n* = 20), Behcet’s Disease (BD) (*n* = 6), and JIA (*n* = 13). Inclusion criteria: patients with rheumatoid arthritis (RA) were selected using the American College of Rheumatology (ACR)/European League Against Rheumatism (EULAR) classification criteria for RA published in 2010 [13]. Disease activity score using 28 joints, erythrocyte sedimentation rate (ESR), and the C-reactive protein level (DAS-28) were utilized to measure the RA activity [14]. On the other hand, Systemic Lupus International Collaborating Clinics Criteria 2012 (SLICC) were used for the diagnosis of systemic lupus erythematosus (SLE) [15], whereas the disease activity was evaluated according to the SLEDAI 2K score [16]. Patients with psoriatic arthritis and Behcet’s disease were selected according to the Psoriasis Area and Severity Index (PASI) [17] and Behcet’s Disease International Clinical Criteria [18], respectively. Activity in patients with psoriatic arthritis was measured using the PSA Disease Activity Score (PASDAS) [19]. Behcet’s disease activity was evaluated by the Behçet’s Disease Current Activity Form (BDCAF) [20]. Additionally, the Juvenile Arthritis Disease Activity Score (JADAS) was used to evaluate the disease activity in JIA patients [21]. An additional 85 apparently healthy age and sex matched volunteers were included as a control group. Volunteers were selected based on the criterion that they were healthy at the time of sampling.

### Detection of Peripheral Blood sjTRECs

Blood samples of two milliliters of peripheral blood were collected from each participant into EDTA vacutainers and stored at −80 °C until further processing.

DNA was extracted according to the manufacturer’s instructions, and genomic DNA was extracted from the blood samples using a QIAamp DNA blood mini kit (Qiagen, Hilden, Germany). Following extraction, the DNA concentration was determined, in each sample, by a NanoDrop One Spectrophotometer (Thermo Scientific, Waltham, MA, USA).

Measuring *sjTRECs* by qPCR peripheral *sjTRECs* levels was assessed by qPCR, using QuantiTect SYBR Green PCR kit (Qiagen, Germany), by the StepOnePlus Real-Time PCR System (Applied Biosystems, Foster City, CA, USA), according to the manufacturer’s instructions. Real-time PCR was performed using 40 ng DNA in 25μL reaction volumes, containing 400 nM of each primer. PCR conditions were 95 °C for 15 min (initial heat activation), then 94 °C for 15 sec (denaturation), 54 °C for 30 sec (annealing), and 72 °C for 20 sec (extension), for 45 cycles. Specific primer pairs were used to amplify the *sjTREC* (accession no. NT_026437) and *TATA box Binding Protein* (*TBP*) (accession no. NG_008165). Primer sequences were as follows: *sjTREC* forward: 5′-CCA TGC TGA CAC CTC TGG TT-3′, *sjTREC* reverse: 5′-TCG TGA GAA CGG TGA ATG AAG-3′ [3], TBP forward: 5′-TTAGCTGGCTCTGAGTATGAATAAC-3′, and *TBP* reverse: 5′-AGCTGAAAACCCAACTTCTGT-3′ [22]. The level of *sjTRECs* in each sample was determined after correction by *TBP* using the dCT method (dCt value: dCt = CtTBP − Ct sjTREC) [23]. The analysis of the melt curves was accomplished using default setting temperature. The *sjTRECs* and *TBP* melting temperatures were around 83 °C and 76.5 °C, respectively. PCR reactions were conducted in duplicate for each sample, and average values were used for data analysis. Non-template controls were used in each run to ensure the absence of non-specific amplification for both genes throughout the entire work.

Statistical analysis via the computerized statistical package STATA/SE version 11.2 for Windows (STATA Corporation, College Station, Texas), and MS Excel were used for data entry, presentation, and analysis. Descriptive statistics, mean ± standard deviation (SD), range, frequency, and percentage were used to summarize data as appropriate. The Shapiro-Wilk W test was used to examine the distribution of numerical data. Categorical data were compared using the Chi-square test and the Student’s *t*-test (*t*) and the One-Way Analysis of Variance (ANOVA; F) to detect differences in dCt (CtTBP−CtsjTREC) values between the study groups, as appropriate. The Pearson correlation coefficient (r) was used to evaluate the correlation between dCt values and chronological age. Linear prediction models of chronological age conditioned on dCt values were developed for the different groups. The correlation between predicted ages and chronological ages were tested using the Pearson correlation coefficient (r), and the mean absolute deviation (MAD) of predicted ages from chronological ages were calculated. Statistical significance was accepted at *p* < 0.05.

## 3. Results

### 3.1. Study Population

Table 1 shows comparisons between the three study groups regarding gender and age. There were no significant differences between the study groups regarding their gender and age distributions (*p* > 0.05).

### 3.2. sjTREC as a Marker of Early Immunosenescence in Autoimmune and COVID 19 Patients

**Figure 1:**
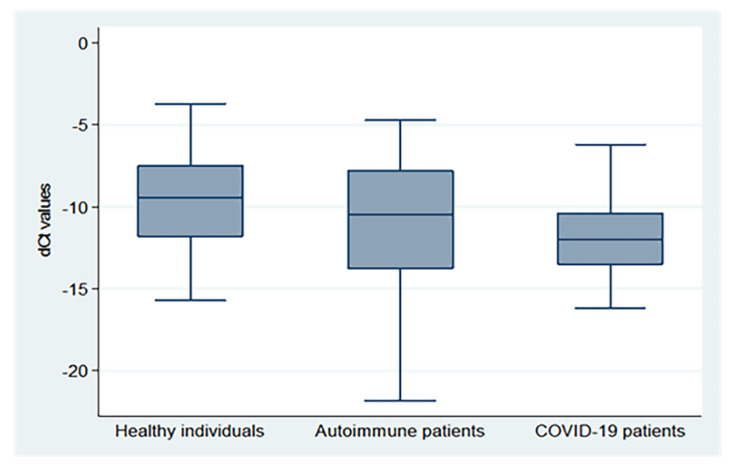
dCt (CtTBP−CtsjTREC) values for healthy individuals, autoimmune patients, and COVID-19 patients.

There were significant differences in the mean dCt values between the three groups (*p* = 0.0002), with the highest values in healthy subjects, followed by autoimmune and COVID-19 patients (−9.62 ± 2.93, −11.30 ± 4.15, and −11.81 ± 2.52, respectively), as demonstrated in Figure 1 (X^2^: Chi-square test; F: One Way Analysis of Variance (ANOVA)).

### 3.3. Peripheral sjTRECs Levels of the Studied Groups Differ According to the Age Subgroup and Gender

Comparisons of dCt (CtTBP−CtsjTREC) values between the studied healthy individuals, autoimmune patients, and COVID-19 patients of different gender and age group are shown in Table 2. Mean dCt values progressively decreased in older age groups compared to young groups for the three studied groups (*p* < 0.001). There were significant differences in dCt values between healthy individuals, autoimmune patients, and COVID-19 patients for those aged 18–34 and 35–49 years (*p* = 0.004 and *p* = 0.001, respectively). There were no significant differences in dCt values between male and female subjects, except for autoimmune patients (*p* = 0.03).

### 3.4. Peripheral sjTRECs Levels Are Insignificantly Decreased in Severe COVID-19 and Active Auto Immune Cases

**Figure 2:**
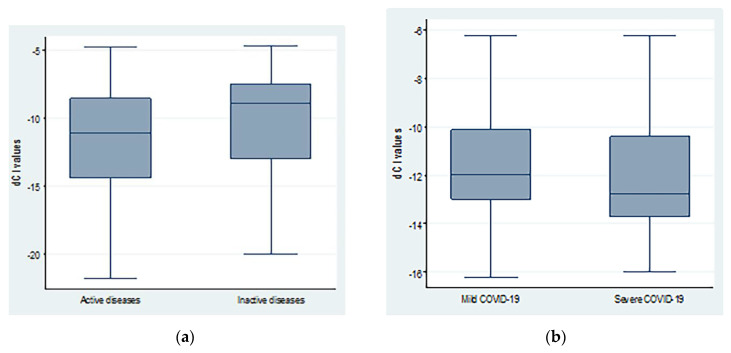
dCt (CtTBP−CtsjTREC) values regarding (**a**) autoimmune diseases activity and (**b**) COVID-19 severity.

The average dCt values were lower in patients with severe COVID-19 compared to mild disease. However, the difference was not statistically significant (−11.41 ± 2.69 vs. −12.29 ± 2.25; *p* = 0.20). Similarly, patients with active autoimmune diseases showed non-significant lower dCt values (−11.68 ± 4.22 vs. −10.26 ± 3.85; *p* = 0.15) when compared to inactive cases, as shown in Figure 2.

### 3.5. Peripheral sjTRECs Levels Had Significant Negative Correlation with Chronological Age in All Studied Groups

**Figure 3:**
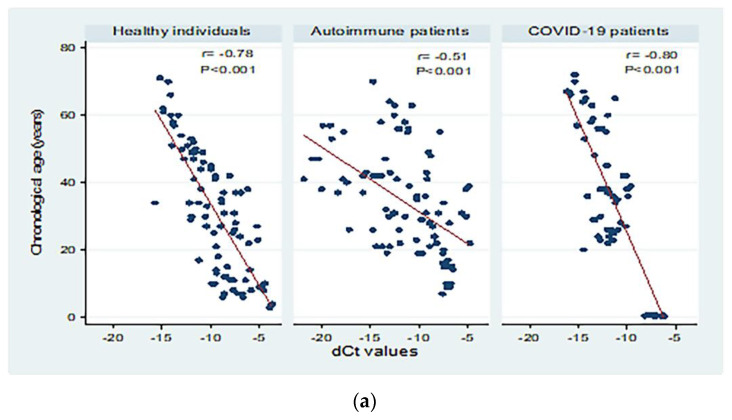

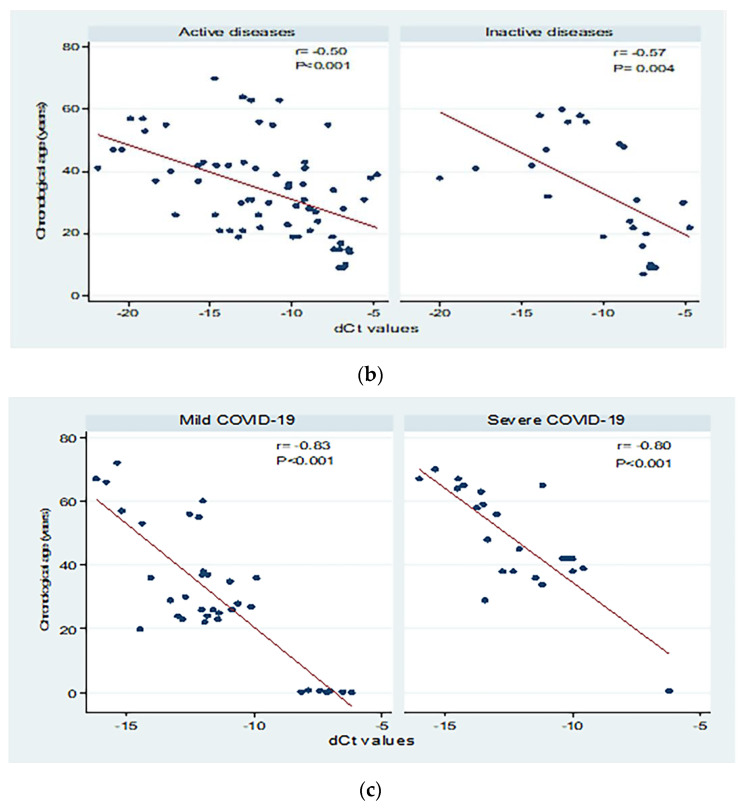
Correlations between chronological age (in years) and dCt (CtTBP−CtsjTREC) values among healthy individuals, autoimmune patients, and COVID-19 patients (**a**), patients with active and inactive autoimmune diseases (**b**), and COVID-19 patients with different disease severity (**c**). r: Pearson correlation coefficient; *p*: probability, significant correlations at *p* < 0.05.

Figure 3 shows correlations between chronological age (in years) and dCt values for the different study groups. There were moderate to high significant negative correlations between chronological age and dCt values (*p* < 0.001; Panel a). Significant moderate negative correlations were detected in patients with both active and inactive autoimmune diseases (*p* < 0.001 and *p* = 0.004, respectively, Panel b). The correlation between chronological age and dCt values was negative, and high correlation was observed in patients with mild and severe COVID-19 (correlation coefficient = −0.84; 0.80 respectively, *p* < 0.001; Panel c).

### 3.6. Peripheral sjTRECs Levels as a Biomarker for Age Prediction in All Studied Groups

Table 3 shows linear prediction models for chronological age conditioned on dCt values in the different study groups. The R-square (R^2^) value ranged from 0.26 in autoimmune patients to 0.61 and 0.64 for healthy individuals and COVID-19 patients, respectively.

**Figure 4:**
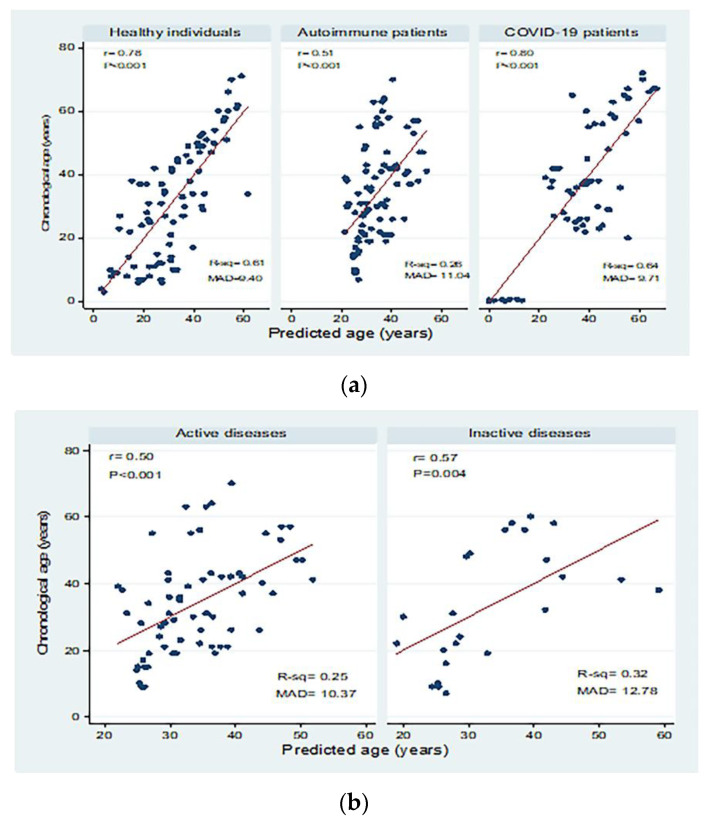

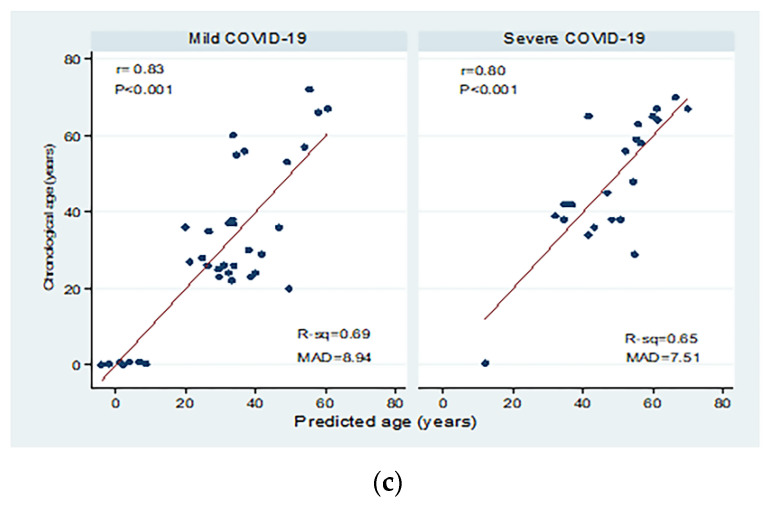
Correlations between chronological age (in years) and predicted age (in years) in (**a**) healthy individuals, autoimmune patients, and COVID-19 patients, (**b**) patients with active and inactive autoimmune diseases, and (**c**) COVID-19 patients with different disease severity. r: Pearson correlation coefficient; *p*: probability, significant correlations at *p* < 0.05; R-sq: R square; MAD: mean absolute deviation.

Correlations between chronological age (in years) and predicted age (in years) in the different study groups are shown in Figure 4. There were significant moderate to high positive correlations between chronological age and predicted age for healthy individuals, autoimmune patients, and COVID-19 patients with mean absolute deviations (MAD) of 9.40, 11.04, and 9.71, respectively. Moderate correlations were detected in patients with active and inactive autoimmune diseases, with MAD = 10.37 and 12.78, respectively. There were significant positive correlations between chronological and predicted ages in COVID-19 patients with mild disease (correlation coefficient = 0.84; *p* < 0.001; MAD = 8.79) and severe disease (correlation coefficient = 0.80; *p* < 0.001; MAD = 7.51).

Comparisons of the age, sex distribution, and the *sjTRECs* dCt values between RA, SLE, and healthy individuals revealed that there were significant differences as regards age groups (*p* < 0.001). Additionally, significant differences in dCt values were noticed between healthy individuals and RA and SLE patients, with the highest values in healthy individuals and the least values in RA patients (−9.62 ± 2.93, −10.88 ± 4.85, and −12.80 ± 2.54; *p* = 0.0006). Data are shown in supplementary material as Table S1.

The correlation of *sjTRECs* levels with laboratory data of autoimmune patients was studied, and a significant negative correlation between dCt values and anti dsDNA (r = −0.37; *p* = 0.04) was detected. Disease duration in patients with autoimmune disorders ranges between six months and 16 years. Insignificant correlation (*p* = 0.17) was found between dCt values and the autoimmune disease duration, as seen in supplementary material Table S2.

## 4. Discussion

Age markers can be evaluated by looking at how they react to different diseases and environmental conditions that might impair the accuracy of calendar age prediction. In prediction models intended to estimate chronological age, it is not recommended to use such markers, which are altered by the environment [24]

Our study population (healthy group, patients with autoimmune diseases, and COVID-19 patients) has been categorized into four age groups (sub-adults, young adults, middle age, older age). Such stratification revealed that *sjTRECs* expression was significantly decreased due to age progression across the older age groups in cases and controls (*p* < 0.001). Data from a previous report [25] supported the notion that defective central tolerogenic function of the thymus is a fundamental aspect in the pathophysiology of autoimmune and virus-related diseases, including COVID-19, which may suggest a likelihood association between the immunopathogenesis of autoimmune diseases and COVID-19.

In the current study, all the sub-adults with autoimmune diseases were diagnosed as JIA patients. It was previously reported that the thymic function in children with JIA is equivalent to that of the healthy control children [26]. A finding was parallel to our results that showed insignificant differences in *sjTRECs* levels between the three studied subadults’ groups < 18 years (*p* = 0.93). The ability of the thymus to replace the apoptotic T lymphocytes is not consistent across the older patients. In children, the effective thymic activity and T cell functions defend them against viral infections, autoimmune disorders, and malignancies. They also play critical roles in SARS-CoV-2 pathogenesis. It was hypothesized that the thymus’s and T cells’ functions defend the children from the SARS-CoV-2 effects [27].

The latest three coronavirus epidemics were reported with an increased mortality in adults, but, SARS-CoV and MERS-CoV have not caused deaths in infected children, and it was concluded that children usually presented with mild coronavirus infections, including SARS-CoV-2 infection [28].

The dCT levels of *sjTRECs* in young adults and middle age groups with autoimmune disorders and COVID 19 were found to be highly significantly lower than the corresponding control groups (*p* = 0.004, 0.001 respectively). This can be explained by different studies on adults with autoimmune disorders that revealed a reduction in *sjTRECs* counts, suggesting that the disturbances in *sjTRECs* dynamics represent a pivotal component in autoimmune disease pathophysiology [29,30,31]. Other studies linked the occurrence of premature thymic atrophy in numerous autoimmune diseases to the pathogenesis of such diseases, and they further related this premature involution of the thymus to the significant alteration in Th1 homeostasis in patients with such diseases [32].

Notably, COVID-19 patients ≥ 18 years included in our study showed reduced *sjTRECs* level when compared to the age matched control group, a relationship which was only significant among the young adults. Such a result may be explained by the peripheral lymphopenia reported in the peripheral circulation of COVID-19 patients [33]. Recent reports also suggested that SARS-CoV can negatively influence the development and migration of thymocytes to other lymphoid tissues [25] and reduce the number of B, T, Th, and NK l cells, which has also been observed in SARS-CoV-2 infected patients [34]. Peripheral lymphopenia may be the result of immune-mediated lymphocyte damage, apoptosis, or inhibition of the bone marrow or thymus [35]. Yet, a different study has observed an increased thymus gland size associated with increased T lymphocytes production as an adaptive compensatory mechanism for the lymphopenia induced by SARS-CoV-2 infection [36].

The clinical manifestations seen in COVID-19 patients above the age of 50 years can be attributed to the uncontrollable, weak antiviral response due to immunosenescence and thymic involution [27]. Elderly COVID-19 patients have deficient thymic function, which may lead to poor disease prognosis in this age group [36]. Consequently, defective thymic adaptation in older COVID-19 patients may explain our results among older patients ≥ 50 years, where the *sjTRECs* levels were nearly matching each other’s in the three groups (cases and control) (*p* = 0.88). Immunosenescence due to the ageing process may explain the extreme reduction in the thymic function that results in very low *sjTRECs* level at older age in the three studied groups. Accordingly, inhomogeneity of the thymic function reported in elderly individuals can compromise the accuracy of using *sjTRECs* quantitation for age estimation [37].

The dCT level of *sjTRECs* showed significant negative correlation with chronological age in all the studied groups, including healthy (r = −0.78), COVID-19 (r = −0.80), and autoimmune patients (r = −0.51). Regarding the control group, our results (R^2^ = 0.61) are consistent with those of Ibrahim et al. [9], who studied the same ethnic group and suggested quantification of *sjTRECs* in peripheral blood as an efficient method for estimating age. Their research also found a highly significant negative correlation between *sjTRECs* level and individual age (R^2^ = 0.87). Similar results have been reported by Ou et al. [22] and other authors [3], who found that the *sjTRECs* levels in old bloodstain and blood samples were decreased in an age-dependent manner, with R^2^ = 0.759 and R^2^ = 0.835, respectively. Cho et al. [38] confirmed the linear negative regression curve between *sjTRECs* levels and age, with R^2^ = 0.807. In 2018, Yamanoi et al. [23] studied the *sjTRECs* level using SYBR green PCR, and their results indicated that *sjTRECs* level has declined with age in bloodstains from the Japanese population, with r = −0.786 (R^2^ = 0.617). The minor discrepancies between the results of the diverse studies may be due to the differences in the sample size, genetic, medical, as well as environmental factors [9,22]. Other contributing factors that can affect *sjTRECs* level include variable ethnic groups, using different age groups, storing the samples for a long period, and the detection procedures [39].

In our study, insignificant differences were detected in the mean levels of *sjTRECs* between the two genders in both healthy and COVID-19 subjects (*p* = 0.40, 0.67 respectively). This result is consistent with the results of Ibrahim et al. [9] and Ou et al. [22], who had reported no differences in *sjTRECs* levels between males and females, in healthy Egyptian and Chinese populations, respectively. Data collected from other studies [40,41] have publicized that men have lower average copy numbers of *sjTRECs* than women. Other authors [3] have also reported a significant effect of gender on *sjTRECs* levels, and they suggested ignoring this effect, since its impact on the predicted age values is minor. Interestingly, our study has identified a significant reduction (*p* = 0.03) in the mean levels of *sjTRECs* in female patients with autoimmune disorders when compared to the males of the same group. It is unclear why women have reduced *sjTRECs* levels, although the majority of the published studies showed either no sex differences or reduced *sjTRECs* in men. Yet, it may be attributed to differences in patients’ characteristics. Moreover, women are known to be at higher risk of developing autoimmune disorders [42].

It was also reported that *TREC* levels are always higher in the CD8^+^ T-cell subpopulation compared to CD4^+^ T cells. Besides, previous analysis of data extracted from patients with RA, SLE, psoriatic arthritis, and JIA suggested enrichment and activation of CD4^+^ T cell subpopulation during disease pathogenesis [43,44,45,46]. The XX sex chromosome pair is recognized as disease promoting chromosomes, as matched to the XY-pair in psoriasis [42]. Our study includes patients with diverse autoimmune disorders with different immune reactants. The sum of *sjTRECs* level detected could also be influenced by the different effects of sex hormones and sex chromosomes on the immunopathogenesis of such diseases. Whether the course of the autoimmune disease is influenced by sex difference or not is still controversial and needs further investigations to identify the distinct influence of gender on the pathogenesis of each autoimmune disease.

Disease activity in patients with autoimmune disorders didn’t affect the levels of *sjTRECs* significantly, and the *sjTRECs* expression didn’t show any significant correlation with disease activity among the studied cases. Similarly, Kurosaka et al.’s [47] results observed an insignificant correlation between *sjTRECs* level and SLE disease activity. However, our results revealed lower *sjTRECs* levels in the peripheral blood of patients with active disease, which is consistent with Vieira et al. [48], whose work revealed an inverse correlation between SLEDAI score and *sjTRECs* counts in CD8^+^, but not in CD4^+^ T lymphocytes. Fascinatingly, patients with inactive SLE displayed similar *sjTRECs* level to healthy controls in CD4^+^ and CD8^+^ T cells [48]. We may attribute low *sjTRECs* level in patients with active autoimmune diseases, such as SLE, to many factors; one of them is the extremely high doses of immunosuppressive drugs and glucocorticosteroids which are well-known to persuade thymus atrophy and to suppress lymphopoiesis [49]. It has been revealed that many T cell dysfunctions in SLE are more noticeable in patients with active disease [50]. It is also known that patients with RA show such signs for an exhausted immune system. Osborne et al. [51] revealed, in their study, that the baseline counts of *sjTRECs* were inversely correlated with the activity of RA. However, the correlation was absent with subsequent follow-up and disease progression.

The average dCt values were insignificantly lower in patients with severe COVID-19 compared to mild disease (*p* = 0.20). The chronological age and dCt values were correlated in COVID-19 patients, and we found a high negative correlation with R^2^ = −0.84; 0.80 in mild and severe cases, respectively (*p* < 0.001). Patients with severe COVID-19 are manifested by lymphopenia, particularly T cell loss [52]. CD 26 T cells apoptosis is the principal mechanism responsible for such lymphopenia. SARS-CoV-2 spike protein targets the cells’ CD 26 T, resulting in an impaired immune system [53,54]. In the elderly, immunosenescence and inflammaging are proposed as high-risk factors for severe COVID-19, implying that the age-related clinical severity of COVID-19 is due to diminished antiviral function and increased self-damaging effects of the immune system [55,56].

The use of the age prediction model to assess the difference between predicted and chronological age among the studied healthy and patient groups is crucial. Data obtained from the control group were used to create the following age prediction equation:

Y = −14.89–4.85 X ± 11.42, where the 11.42 refers to the SE and R^2^ = 0.61. The equation was utilized to compare the predicted and chronological age in healthy individuals. However, in the model for patients with various autoimmune disorders, the equation for age prediction showed a lower value (Y = 12.25–1.91 X ±13.47), R^2^ = 0.26. While for COVID-19 patients, the equation was more or less near to the healthy group (Y = −41.63–6.68 X ± 12.60), R^2^ = 0.64. The current results indicate that COVID-19 may affect the immune age and thymic function significantly in young adult patients, though the age prediction model does not show such a relationship. The MADs from the chronological age were 9.4, 11.04, and 9.71 years in healthy, autoimmune, and COVID-19 subjects, respectively, with a higher deviation in autoimmune patients when compared to the other two groups. Therefore, it was found that the accuracy of age prediction decreases with higher deviation as an impact of various autoimmune disorders. At older age groups, *sjTRECs* levels were very low and, thus, *sjTRECs* levels in older patients with COVID-19 and autoimmune diseases match the controls with the same predicted age. While in patients at the age of 50 and younger, *sjTERCs* levels in patients with autoimmune diseases match those of older age by around 11.04 years.

This obvious age reduction in autoimmune patients could be attributed to the alteration of T cell dynamics in RA cases and, possibly, to increased T cells’ turnover as part of the RA disease pathogenesis. Koetz et al. [30] stated that the age-dependent decline in the number of *sjTREC*-containing CD4^+^ T cells was not different in the RA patients compared to the healthy individuals; however, the curves were shifted by approximately 20 years towards a younger age. Therefore, RA patients aged 20–30 years old have *sjTRECs* level equivalent to healthy individuals of 50–60 years old. Similarly, *sjTRECs* level was significantly lower in SLE patients, which supports the idea that T cell subset imbalance and aberrant expression of the key signaling molecules on their surfaces contribute significantly to SLE disease pathogenesis [57]. A limitation to our study includes the small number of autoimmune patients’ subgroups, which impedes studying the effects of each individual disease on *sjTRECs* levels. The follow up process of the recovered COVID-19 patients during the peak of the pandemic and the economic burden was another limitation to study its effect during the convalescence stage.

## 5. Conclusions

Autoimmune diseases have dramatically reduced the levels of *sjTRECs* in the peripheral blood of Egyptian patients, particularly young adults and middle-aged individuals. Such a finding may affect the reliability of using *sjTRECs* alone as a marker for age estimation in patients with such chronic immunologic disorders. It is recommended to couple *sjTRECs* with other age estimate indicators for forensic purposes. Acute infection with SARS-CoV-2 in young adults has a substantial negative influence on the thymic function. Nevertheless, the MAD and the age prediction equations of all COVID-19 patients were more or less near to the healthy individuals, which may oppose the notion that COVID-19 may implicate the age prediction using *sjTRECs*. Measuring *sjTRECs* level is an indicator of thymic function and immunosenescence. Consequently, we can also infer that the immune system of the affected age groups also shows signs of an early immunosenescence due to the effect of the disease pathogenesis on the T cell function. Above the age of 50, the marked reduction in *sjTRECs* levels, and the noticeable similarity in the low thymic functions among the three studied groups, reveal that age-related involution of the thymus and immunosenescence starts around the age of 50 years in the healthy Egyptian population. Though autoimmune disease activity and COVID-19 severity showed significant negative correlation with age, they have slightly reduced the *sjTRECs* levels as compared to inactive autoimmune and mild COVID-19 cases.

Finally, we believe that further longitudinal study is required to assess the dynamic effects of the individual autoimmune diseases and COVID-19 on *sjTRECs* and the thymic function in patients’ groups and subgroups by using larger sample size. Using other markers, in addition to *sjTRECs* such as epigenetic markers, e.g., microRNA (miRNA), may provide insights to comprehend how genes control and maintain the microenvironment of the thymus during the ageing process.

## Figures and Tables

**Table 1:** Characteristics of the studied groups.

		Healthy Individuals (*n* = 85)	Autoimmune Patients (*n* = 90)	COVID-19 Patients (*n* = 58)	Test	*p*
		*n* (%)	*n* (%)	*n* (%)		
Gender	Females	49 (57.65)	54 (60.00)	27 (46.55)	X^2^ = 2.77	0.25
	Males	36 (42.35)	36 (40.00)	31 (53.45)		
Age (years)	Subadults (<18)	25 (29.41)	13 (14.44)	8 (13.79)	X^2^ = 12.31	0.06
	Young adults (18–34)	23 (27.06)	35 (38.89)	16 (27.59)		
	Middle age (35–49)	21 (24.71)	26 (28.89)	16 (27.59)		
	Older age ≥ 50	16 (18.82)	16 (17.78)	18 (31.03)		
	Mean ± SD Range	31.79 ± 18.19 3–71	33.88 ± 15.57 7–70	37.35 ± 20.97 0.11–72	F = 1.65	0.19

X^2^: Chi-square test; F: One Way Analysis of Variance (ANOVA).

**Table 2:** Comparisons of *sjTRECs* levels (mean dCt values) between the healthy group, autoimmune patients, and COVID-19 patients, according to their gender and age.

		Healthy Individuals		Autoimmune Patients		COVID-19 Patients		F	*p*
		*n*	Mean ± SD	*n*	Mean ± SD	*n*	Mean ± SD		
Age group (years)	Subadults (<18)	25	−7.23 ± 2.06	13	−7.04 ± 0.36	8	−7.07 ± 0.73	0.08	0.93
	Young adults (18–34)	23	−9.26 ± 2.48 !	35	−10.14 ± 2.91 !	16	−12.04 ± 1.16 ^ab^!	5.85	0.004
	Middle age (35–49)	21	−9.86 ± 1.92 !	26	−13.54 ± 4.73 ^a^!¶	16	−11.42 ± 1.34 !	7.24	0.001
	Older age ≥ 50	16	−13.52 ± 1.13 !¶‡	16	−13.67 ± 3.49 !¶	18	−14.07 ± 1.47 !¶‡	0.13	0.88
	F	31.79		13.97		56.54			
	*p*	<0.001		<0.001		<0.001			
Gender	Females	49	−9.39 ± 2.68	54	−12.07 ± 4.33 ^a^	27	−11.66 ± 2.91 ^a^	8.18	0.0005
	Males	36	−9.93 ± 3.25	36	−10.15 ± 3.64	31	−11.95 ± 2.16 ^a^	4.09	0.02
	*t*	0.84		2.20		0.42			
	*p*	0.40		0.03		0.67			

F: One Way Analysis Of Variance (ANOVA), *t*: Student’s *t*-test, *p*: probability, statistically significant at *p* < 0.05, a: significant difference compared to healthy individuals, b: significant difference compared to autoimmune patients, !: significant difference compared to subadults, ¶: significant difference compared to young adults, ‡: significant difference compared to middle-ages.

**Table 3:** Chronological age prediction conditioned on dCt (CtTBP−CtsjTREC) values in the different study groups.

Group		N	*p*	R^2^	Adj. R^2^	SE	MAD	B (95% CI)	Equation
Healthy individuals		85	<0.001	0.61	0.60	11.42	9.40	−4.85 (−5.70 to −4.01)	Y = −14.89 − 4.85X
Autoimmune patients	Active	66	<0.001	0.25	0.24	12.99	10.37	−1.75 (−2.51 to −0.99)	Y = 13.54 − 1.75X
	Inactive	24	0.004	0.32	0.29	14.89	12.78	−2.62 (−4.29 to −0.95)	Y = 6.61 − 2.62X
	Total	90	<0.001	0.26	0.25	13.47	11.04	−1.91 (−2.60 to −1.23)	Y = 12.25 − 1.91X
COVID-19 patients	Mild	35	<0.001	0.69	0.68	11.69	8.94	−6.48 (−8.01 to −4.95)	Y = −44.24 − 6.48X
	Severe	23	<0.001	0.65	0.63	10.05	7.51	−5.91 (−7.89 to −3.93)	Y = −24.56 − 5.91X
	Total	58	<0.001	0.64	0.64	12.60	9.71	−6.68 (−8.01 to −5.36)	Y = −41.63 − 6.68X

R^2^: R-square; Adj. R^2^: adjusted R-square; SE: standard error; MAD: mean absolute deviation; B: regression coefficient; CI confidence interval; Y: predicted age; X: *sjTRECs*.

## Data Availability

Not applicable.

## Supplementary Materials

The following supporting information can be downloaded at: https://www.mdpi.com/article/10.3390/biomedicines10123193/s1, Table S1: Comparisons of *sjTRECs* dCt levels between RA, SLE and healthy individuals; Table S2: Correlation between dCT levels of *sjTRECs* and the laboratory data/disease duration in autoimmune patients.
