# Supplementary material for: Exploiting Signal Joint T Cell Receptor Excision Circle to Investigate the Impact of COVID-19 and Autoimmune Diseases on Age Prediction and Immunosenescence
Source: Biomedicines. 2022 Dec 9;10(12):3193. doi: 10.3390/biomedicines10123193 (PMC9775389; doi:10.3390/biomedicines10123193)
Supplement: Supplementary file 1 [file biomedicines-10-03193-s001.zip › biomedicines-2061647-supplementary.pdf]

Supplementary Material

(Manuscript ID: biomedicines-2061647)

Table S1. Comparisons of *sjTRECs* dCt levels between RA, SLE and healthy individuals

Characteristic		Healthy individuals (no.=85)		RA patients (no.=21)		SLE (no.=30)		Test	P
		No.	%	No.	%	No.	%		
Gender	Females	49	57.65	13	61.90	20	66.67	X^2 = 78	0.68
	Males	36	42.35	8	38.10	10	33.33		
Age (years)	Subadults (<18)	25	29.41	0	0.00	0	0.00	FET	<0.001
	Young adults (18-34)	23	27.06	8	38.10	21	70.00		
	Middle age (35-49)	21	24.71	7	33.33	7	23.33		
	Older age (50->60)	16	18.82	6	28.57	2	6.67		
	Mean ±SD<br>Range	31.79±18.19<br>3-71		40.81±13.86<br>21-70		30.93±9.15<br>19-55		F = 2.99	0.05
dCt values	Mean ±SD<br>Range	-9.62±2.93<br>(-15.72)-(-3.71)		-12.80±2.54[a]<br>(-18.98)-(-9.21)		-10.88±4.85<br>(-21.84)-(-4.72)		F=7.79	0.0006

X^2: Chi-square test; FET: Fisher Exact Test; F: One Way Analysis Of Variance (ANOVA)
P: Probability, statistically significant at P<0.05
a: significant difference compared to healthy individuals

Table S2. Correlation between dCT levels of *sjTRECs* and the laboratory data / disease duration in autoimmune patients

	No.	Mean ±SD<br>Range	dCt (Ct_{TBP}–Ct_{sjTREC}) values	
			r	P
**ESR**	90	44.42±20.54<br>8-90	-0.12	0.25
**CRP**	90	25.27±17.73<br>5-60	-0.13	0.23
**RF (U/ml)**	17	70.59±26.37<br>32-120	-0.14	0.58
**ACPA**	19	39.6±35.48<br>0.5-100	-0.17	0.47
**C3**	30	86.9±20.49<br>11-117	-0.11	0.56
**C4**	30	11.01±5.13<br>3-19.2	-0.09	0.63
**Anti dsDNA**	30	58.85±20.24<br>12.2-98.2	**-0.37**	**0.04**
**Disease duration (years)**	90	4.80±3.32<br>0.5-16	-0.15	0.17

r: Pearson correlation coefficient; P: Probability, significant correlations at P<0.05.
